# Assessing performance of simplified bioassays for soil-borne pathogens in smallholder systems of western Kenya

**DOI:** 10.3389/fpls.2024.1389285

**Published:** 2024-08-15

**Authors:** Joyce C. Mutai, Jane E. Stewart, Beth Medvecky, John T. Dobbs, Steven J. Vanek, John Ojiem, Gabriel Chege, Steven J. Fonte

**Affiliations:** ^1^ Department of Soil and Crop Sciences, Colorado State University, Fort Collins, CO, United States; ^2^ Department of Agricultural Biology, Colorado State University, Fort Collins, CO, United States; ^3^ Innovations in Development, Education and the Mathematical Sciences (IDEMS) International, Reading, United Kingdom; ^4^ Kenya Agricultural & Livestock Research Organization, Kibos Centre, Kisumu, Kenya

**Keywords:** *Fusarium*, manure, plant parasitic nematodes, *Pythium*, soil health, synthetic fertilizer

## Abstract

**Introduction:**

Soil-borne pathogens cause considerable crop losses and food insecurity in smallholder systems of sub-Saharan Africa. Soil and crop testing is critical for estimating pathogen inoculum levels and potential for disease development, understanding pathogen interactions with soil nutrient and water limitations, as well as for developing informed soil health and disease management decisions. However, formal laboratory analyses and diagnostic services for pathogens are often out of reach for smallholder farmers due to the high cost of testing and a lack of local laboratories.

**Methods:**

To address this challenge, we assessed the performance of a suite of simplified soil bioassays to screen for plant parasitic nematodes (e.g., *Meloidogyne*, *Pratylenchus*) and other key soil-borne pathogens (*Pythium* and *Fusarium*). We sampled soils from on-farm trials in western Kenya examining the impact of distinct nutrient inputs (organic vs. synthetic) on bean production. Key soil health parameters and common soil-borne pathogens were evaluated using both simple bioassays and formal laboratory methods across eleven farms, each with three nutrient input treatments (66 samples in total).

**Results and Discussion:**

The soil bioassays, which involved counting galls on lettuce roots and lesions on soybean were well correlated with the abundance of gall forming (*Meloidogyne*) and root lesion nematodes (e.g., *Pratylenchus*) recovered in standard laboratory-based extractions. Effectiveness of a *Fusarium* bioassay, involving the counting of lesions on buried bean stems, was verified via sequencing and a pathogenicity test of cultured *Fusarium* strains. Finally, a *Pythium* soil bioassay using selective media clearly distinguished pathogen infestation of soils and infected seeds. When examining management impact on nematode communities, soils amended with manure had fewer plant parasites and considerably more bacterivore and fungivore nematodes compared to soils amended with synthetic N and P. Similarly, *Pythium* presence was 35% lower in soils amended with manure, while the *Fusarium* assays indicated 23% higher *Fusarium* infection in plots with amended manure. Our findings suggest that relatively simple bioassays can be used to help farmers assess soil-borne pathogens in a timely manner, with minimal costs, thus enabling them to make informed decisions on soil health and pathogen management.

## 1 Introduction

Soil-borne pathogens, including plant pathogenic fungi and plant parasitic nematodes (PPN), are significant pests that reduce crop yields worldwide, causing stunting, yellowing, reduced quality and quantity of produce, and sometimes complete crop mortality (Moens et al., 2009; George et al., 2016; Jimenez-Hernandez et al., 2021). This problem is of particular concern in sub-Saharan Africa (SSA), where pathogens such as *Fusarium*, *Pythium*, root-knot nematodes (*Meloidogyne* spp.) and lesion nematodes (*Pratylenchus* spp.) pose a major threat to crop production due to continuous cultivation, minimal crop rotation, degraded soils, and limited access to pesticides (Nicol et al., 2011).

Throughout East Africa, *Meloidogyne* and *Pratylenchus* species can cause up to 50% yield decline in some fields (Kimenju et al., 2008; Chirchir et al., 2010; Atandi et al., 2017; Maina et al., 2019). Damage by these nematodes can be especially severe when conditions favor multiple generations per growing season, such as low crop diversity, multiple cropping seasons per year, and favorable soil characteristics (Luc et al., 2005; Coyne et al., 2018; Sikora et al., 2018). *Pratylenchus* and *Meloidogyne* generation times can be as short as three weeks, with both groups known for high reproduction rates and tolerance to a wide temperature range (Jones et al., 2013; Maina et al., 2019). While *Pratylenchus* and *Meloidogyne* are among the most important and widespread pests, especially in the tropics, detailed information on their distribution, severity and economic impact remains limited.

Similarly, *Fusarium* and *Pythium* are problematic soil pathogens across SSA, causing significant crop damage and are abundant in agricultural soils (Maina et al., 2015; Papias et al., 2016; Henry et al., 2019). The abundance of these pathogens in agricultural soils is associated with infected plant debris and roots, use of infested seeds, and soil tillage/disturbance, which leads to hyphal fragmentation, propagule dispersal and facilitates pathogen access to nutrients and oxygen (Silvestro et al., 2013; Maina et al., 2015). Continuous cultivation of soils on farms with limited land resources, insufficient nutrient inputs and residue recycling have led to soil organic matter and nutrient decline, poor soil structure and decreased water holding capacity, contributing to plant stress and susceptibility to various pests and diseases. Pathogens, such as *Fusarium*, commonly attack plants under stress, whether caused by abiotic factors like nutrient deficiency, alternating wetting and drying of soil, extreme temperatures, and waterlogged soils or biotic factors like primary damage from other pests. *Fusarium* species also create a challenge due to their widespread geographic distribution, efficient dispersal mechanisms, ability to grow in diverse substrates, and survival in soil for up to 10 years without a host (Pérez-Hernández et al., 2017; Jimenez-Hernandez et al., 2021). In western Kenya and much of SSA, many soils are characterized by low soil pH, which can influence *Pythium* development, as acidic conditions favor formation of their oospores and sporangia, and decrease plant nutrient availability leading to low plant vigor and greater vulnerability to pathogen infection (Kisinyo et al., 2014; Papias et al., 2016).

Despite numerous studies identifying symptoms caused by soil-borne pathogens and nutrient deficiencies, most smallholder farmers rarely associate these symptoms with soil-related problems due to limited knowledge and similarity of pathogen and nutrient stress symptoms (Ngoya et al., 2023). Nematode infections usually lead to unspecific aboveground disease symptoms akin to indicators of plant physiological stress (George et al., 2016). Additionally, when PPN damage is followed by fungal, viral or bacterial plant diseases such as *Fusarium*, *Pythium*, or *Rhizoctonia* that cause secondary infections via nematode-inflicted wounds, disease symptoms can be more adverse and confusing (Al-Hazmi and Al-Nadary, 2015).

Researchers and extension agents often recommend use of synthetic fertilizers and organic soil amendments such as biochar, compost, manure, and plant residues to enhance soil fertility and reduce the incidence and severity of soil-borne pathogens (Hartmann et al., 2015; Rosskopf et al., 2020; Hou et al., 2022). However, soil and environmental conditions are highly variable, so nutrient and organic amendment additions might be expected to have varying impacts (Tittonell et al., 2005). Site specific soil testing is therefore highly relevant in these situations (Nyamasoka-Magonziwa et al., 2020; Mallory et al., 2022) to best understand soil health status and make context-based decisions on how best to improve agroecosystem productivity.

In western Kenya, common nutrient inputs include farmyard manure and synthetic fertilizers, both typically applied at well below the recommended rates, as well as plant residues and compost, if available. Plant residues are commonly used as a forage source, implying recycling to fields as manure, with varying levels of efficiency (Castellanos-Navarrete et al., 2015), or burning of residues which represents a serious loss of soil C additions and N on some farms (Nyamasoka-Magonziwa et al., 2021). These practices can have implications for plant pathogen pressure in these farms. Soil testing, including tests assessing pathogen prevalence in fields, can help motivate changes in nutrient inputs, removal or burning of plant residues after harvest, and improved efficiency of manure management, so that soil pathogen problems are not exacerbated. Soil testing might also influence crop/variety selection, as well crop rotational patterns. Therefore, accessible tools for learning about soil health, including the connections between soil health status and management practices, offer great promise for smallholder farmers and the organizations they engage with and could greatly facilitate improved soil management decisions.

To help farmers better predict and manage potential soil pathogen issues in their fields, this research sought to: 1) assess the performance of previously developed, simplified soil pathogen tests (for *Fusarium*, *Pythium*, *Pratylenchus* and *Meloidogyne*) by comparing them to standard laboratory analyses, and 2) assess the impact of distinct nutrient inputs (organic vs. synthetic) on key soil health parameters, including soil-borne pathogen prevalence using the simplified methods. We hypothesized that the simplified soil pathogen tests would provide a reliable approximation of established laboratory methods and help provide farmers with more accessible methods for evaluating presence of major soil-borne pathogens. Additionally, we hypothesized that organic nutrient inputs would enhance soil health by increasing organic matter and soil pH, both of which in turn would suppress the major soil-borne pathogens.

## 2 Materials and methods

### 2.1 Study site and experimental design

This research was conducted in Nandi County, in western Kenya, at three locations: Kapkerer (Lat. 0.02 N, Long. 34.78 E; 1400-1700 m), Kapsengere (Lat. -0.01 S, Long. 34.75 E; 1400-1600 m), and Koibem (Lat. 0.15 N, Long. 34.97 E; 1700-1900 m). The locations experience mean annual temperatures of 21°C, 23°C, and 17°C, respectively, and two rainy seasons: February to July (long rains) and September to November (short rains). Mean annual rainfall ranges between 1000-2000 mm. Soils are dominated by highly weathered Nitisols (or Oxisols) with generally low pH (Jaetzold, 2007). Farmers in all three locations typically cultivate small plots of land (0.25-5 ha), with maize and beans being the most common crops.

This research was conducted within a field experiment that was established across eleven farms in the three locations in western Kenya in March 2021 (during the long rains season) to evaluate the impact of different soil fertility amendments on soil health and the productivity of common bean (*Phaseolus vulgaris*). The experiment considered different types of organic matter input such as crop residues, farmyard manure (FYM), and biochar, as well as synthetic fertilizers, and a non-amended control. Treatments were established within 3 x 3 m plots, with each treatment present in two replicate blocks per farm, in a randomized complete block design. For this study, only a sub-set of the treatments were considered: 1) FYM applied at a rate of 5.6 Mg ha^-1^ per season; 2) synthetic fertilizer, 36 kg N ha^-1^ and 92 kg P_2_O_5_ ha^-1^ per season applied as di-ammonium phosphate (DAP); and 3) a control treatment, with no amendment applied.

At planting, all plots were lightly tilled by hand using a hoe, and beans (variety KK red 16) were row-planted, at spacing of 45 cm between rows and 10 cm within rows. FYM and DAP were applied in furrows, ensuring they were not in direct contact with bean seeds. Weeding was conducted twice using a hoe, at 21 and 46 days after planting, with no additional pest control practices or irrigation applied.

### 2.2 Soil sampling and analysis

Soil samples were collected during the long rains season, in late May of 2022, shortly before bean flowering. In each plot, 10 sub-samples were taken to a depth of 15 cm using a soil auger (4 cm diameter) and mixed to form one composite sample per plot (3-5 kg field moist soil). Additional samples were collected for bulk density and aggregate stability by inserting a sharpened metal cylinder (7 cm diameter) to a depth of 5 cm at two representative points in each plot. The cylinder was inserted vertically into the soil by hand and excavated carefully. Samples from the cylinders were transported to the lab in sealed plastic bags placed within protective containers in a cooler. Upon return to the lab, the composite samples were subdivided for assessment of soil-borne pathogens and a range of physical and chemical soil properties. For the nematode assessment assays, 1 kg sub-samples per plot were taken from the composite sample, placed in sealed plastic bags and stored at 4°C until processing (within 2 days of sampling).

Soil used for assessment of *Pythium* and *Fusarium*, as well as soil physico-chemical analyses were air-dried, and passed through 2 mm sieve, and analyzed using low-cost, rapid assessment methods described by Nyamasoka-Magonziwa et al. (2020). Soil pH was measured in a 2:1 deionized water:soil suspension, evaluation of permanganate oxidizable C (POXC) was based on the oxidation of labile soil organic C by potassium permanganate (KMnO_4_), available P was determined using a modified Olsen method, while particulate organic matter (POM) was determined using density flotation with deionized water (Nyamasoka-Magonziwa et al., 2020; also see https://smallholder-sha.org). Additionally, sub-samples were sent to a commercial laboratory in Nairobi for analysis of total soil C and soil texture, using dry combustion and particle size analysis by hydrometer method, respectively.

The bulk density samples were weighed, and a representative sub-sample of ca. 40 g was dried at 105°C to determine moisture content and calculate oven-dry soil mass and bulk density. The remaining field moist soil was carefully passed through an 8-mm sieve and air-dried. A 70 g sub-sample of 8-mm sieved soil was used for evaluation of aggregate stability via a wet sieving method adapted from Elliott (1986). The sample was submerged for 5 minutes, for slaking, and then sieved through a 2 mm and then a 250 μm sieve by carefully lifting the sieve in and out of a pan of water, for a total of 50 oscillations over a 2-minute period. The soil remaining on each sieve was collected, dried at 105°C, and weighed to generate three aggregate size classes (>2000 μm, 250–2,000 μm, < 250 μm). Aggregate stability was calculated as the mean weight diameter (MWD), considered as the fraction of soil mass present in an aggregate size class multiplied by the mean diameter of aggregates in each size class.

### 2.3 Root lesion and root-knot nematode bioassays

To evaluate the disease pressure from root lesion nematodes (*Pratylenchus* spp.) and root-knot nematodes (*Meloidogyne* spp.) in each soil sample, we used Cornell-developed soil bioassays, modified from Gugino et al. (2006; 2008; 2009). Soybean and lettuce plants served as hosts for *Pratylenchus* and *Meloidogyne*, respectively. Two sub-samples (ca. 500 g each) of refrigerated soil from each plot were placed in separate pots (8 cm diameter, 12 cm high, with drainage holes). Three soybean seeds were planted in each pot for the root lesion nematode bioassay, and three lettuce seeds for the root-knot nematode bioassay. Pots were maintained in the greenhouse and watered as needed, with sterile soilless media used as a control.

Three weeks post-germination, the plants were carefully removed, and roots were washed free of soil. Soybean roots were examined for elongated dark brown lesions caused by lesion nematodes, while lettuce roots were examined for root galls caused by root-knot nematodes. The total number of lesions and galls on the entire root system of the three plants in each pot was recorded. Fresh biomass of the cleaned roots was also measured, and the roots were kept cool until subsequent nematode extraction.

Although the number of lesions and root galls was counted in all soybean and lettuce roots, respectively, it was assumed that severely damaged roots could have lower counts due to partial decomposition, making nematode damage less detectable. Therefore, the number of lesions and galls assessed in the bioassay was normalized to the root biomass to account for variability in plant growth due to nematode infestation.

### 2.4 Laboratory extraction of nematodes from plant roots and soil

To verify the interpretation of data collected from the bioassays described above (i.e., lesion or gall counts), which were developed by Cornell researchers as a way of engaging growers, we evaluated nematodes in the roots of soybean and lettuce, and all nematodes (including the free-living taxa) in the soil. Within two days of root harvest, all roots from each pot were macerated in a domestic blender (at about 12000-RPM for 30 s), or chopped with a knife (when roots were too small to use a blender), for extraction of PPN using a modified Hemming Tray method (Bell and Watson, 2001). Bowls were placed on a flat surface and mesh (lined with paper towel filters) was suspended slightly above the bottom of the bowls. The chopped plant root material from each pot was placed in a clean mesh, and partially submerged in water, so that the mesh was in contact with the water. The mesh was covered with a lid and after 48 hours, live nematodes were presumed to have left the plant tissue, passed through the mesh, and then sunk to the bottom of the bowl. Nematodes were transferred into a beaker, left to settle, then passed through a 38-μm sieve. The sieve was carefully rinsed using a spray bottle to ensure all nematodes were captured and then poured into 10 ml vials. Nematodes were then preserved in formalin prior to examination and counting on a slide under a dissecting microscope (10 to 40x magnification power). Specimens were transferred using a handling needle to a microscope slide as needed, for inspection at higher magnification with a compound microscope. All nematodes present were identified to genus level via examination of morphology.

Nematode assessment of soils similarly relied on a modified Hemming Tray extraction method. Deep plates were placed on a flat surface, and then a colander was placed on top of each. Wet filter papers were carefully placed on the sieves ensuring full coverage of the sieves and no wrinkles or air-bubbles. Roughly 200 g of fresh soil from the pots used in the lesion nematode bioassay (with soybean) was placed in each sieve and spread evenly before covering with a lid and placing them on plates containing ca. 200 ml tap water. A spray bottle was used to slowly add water to ensure adequate contact between the sieve and the plate, while being careful not to totally submerge soil. After 48 hours, nematodes that had migrated through the soil down to the plates were rinsed into a beaker, the screen and plate were thoroughly rinsed with a spray bottle for collection. Nematodes were allowed to settle out to the bottom of the beaker for ca. 3 hours, then each sample was passed through a 38-μm sieve and nematodes on the sieve were rinsed into 10 ml vials. Nematodes were then preserved using formalin to ensure intact specimens for identification, as described above.

### 2.5 *Pythium* and *Fusarium* assessment

To assess *Pythium* and *Fusarium* infections, we adapted established bioassay procedures. For *Pythium* assessment, the procedure by Ali-Shtayeh (1986) was followed. Plastic containers were filled with 125 g of soil and brought to 75% water holding capacity. Ten bean seeds were buried in the moistened soil, the container lid was sealed with parafilm, and incubated in the dark at 21°C for 3 days. After incubation, seeds were removed, and the number and rate of rotting seeds were recorded. Seeds were then rinsed with deionized water, blotted dry, placed on a *Pythium*-selective medium (PARP: pimaricin + ampicillin + rifampicin + pentachloronitrobenzene [PCNB] agar) developed by Jeffers and Martin (1986), and incubated for three more days at 21°C. The presence or absence of mycelia growing into the media from individual seeds and the number of colonies were recorded. Soil known to contain *Pythium* inoculum served as a positive control, and autoclaved sand as a negative control. A compound microscope was used to verify *Pythium* by checking for sporangia, antheridia, oogonia, and zoospores. The efficacy of the medium, visual observation of seeds rotting and the mold-colonies growing from individual seeds, and lab-microscope positive identification of *Pythium* provide high confidence for the assay, thus avoiding the need for further validation.

**Figure 1:**
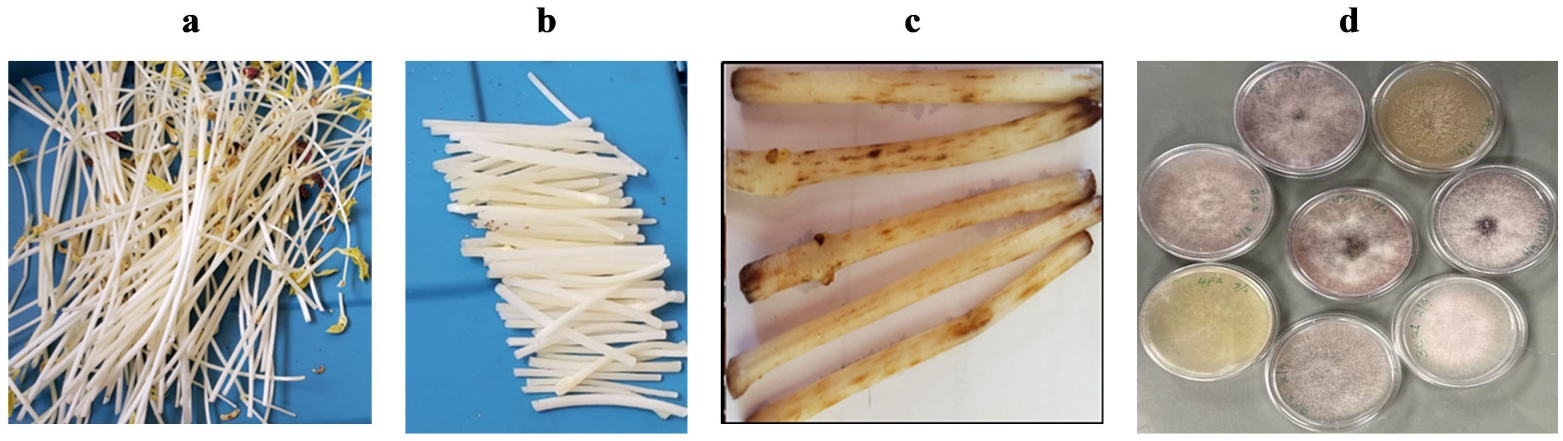
*Fusarium* assessment based on the number of lesions produced by the *Fusarium* pathogens on a 6 cm section of bean stem buried and incubated for 4 days at 25°C in soil samples collected on-farm. Figure panels show the following: **(A)** white stems of beans after growing in darkness for 16 days; **(B)** ca. 6 cm long sections of bean stems (to be buried in infested soil); **(C)** lesions development on bean stems 4 days after burying in soil; and **(D)** a sample of cultures isolated from the lesions.

*Fusarium* baiting assays were adapted from Furuya et al. (1999). Bean seeds were planted in soilless media (Cornell peat mix) and grown in the dark at room temperature for 14 days to obtain long white stems (ca. 25 cm) that can easily show signs of *Fusarium* infestation (**Figure 1A**). Stems were harvested and cut into 6 cm segments (**Figure 1B**). Two sub-samples of air-dried, 2-mm sieved soils (500 g each) from each farm were placed in plastic containers with lids (11 cm diameter, 12 cm high) and adjusted to 50% water holding capacity. Ten bean stem segments were evenly spread and buried in each container, then incubated at room temperature (ca. 25°C). After 4 days, segments were removed, rinsed with clean water, and the number of reddish-brown lesions longer than 1 mm consistent with *Fusarium* damage were counted (**Figure 1C**). Lesions originating from separate infection sites were counted individually even if they joined to produce larger lesions. Controls included bean stems buried in autoclaved sand and soil-free Cornell mix, expected to show no lesions.

### 2.6 Isolation, culturing, and identification of *Fusarium*

*Fusarium* species are common soil microbes, most of them being saprotrophs decomposing organic matter. However, depending on *Fusarium* genetic makeup, inoculum density, virulence levels, and host immune system, some species become pathogenic and can cause root and stem rot, damping-off, necrosis, leaf yellowing and wilting in numerous plant species, under favorable conditions. We observed and counted the number of lesions that developed on the 6 cm long bean segments buried in each pot. To better understand the range of *Fusarium* taxa recovered and the potential pathogenicity of the different types, we cultured two replicated samples of lesions collected from each pot for further analysis. *Fusarium* were isolated by cutting off lesions from each of the two representative bean stems that had been buried in soils from the 66 plots (**Figure 1C**). The cut segments were surface sterilized, blotted dry and then plated in fungal culture ½ PDA medium (Potato Dextrose Agar (PDA) mixed with distilled water and agar, cooled to 45°C). The plates were incubated for 7 days at room temperature and then numbers and differentiated types of fungal colonies were counted. Fungal colony types from the total of ca. 132 plates were grouped based on colony color, growth type, colony reverse color, and color of mycelia. Many of the colonies were white cottony with a dark-purple undersurface on ½ PDA and spores were oval to kidney shaped, with three septate spores, while the few other cultures were pink, reddish and purple, and thin and colorless. Based on these characteristics, we sub-cultured 60 colonies on ½ PDA and incubated at room temperature for 14 days (**Figure 1D**). Cultures were molecularly identified using a standard polymerase chain reaction (PCR) approach was used to extract, sequence, and identify 35 unique colonies. Extraction of DNA was done using a genomic DNA kit (Zymo Research Corporation, Irvine, CA, USA) following manufacturer’s instructions. A NanoDrop Spectrophotometry ND-1000 (NanoDrop Technologies, Montchanin, DE, U.S.A.) was used to determine extracted DNA concentration and quality. Extracts were diluted to 10 ng/μl and stored at −20°C until used. Sanger sequencing of the RNA polymerase II second largest subunit (*rpb2*) locus was conducted using methods similar to Dobbs et al. (2023).

Briefly, a 25 μl reaction was used to amplify the *rpb2* locus for each sample consisting of 2 μl each of forward (RPB2-6F: 5’-TGGGGKWTGGTYTGYCCTGC-3’) and reverse (fRPB2-7cR: 5’-CCCATRGCTTGYTTRCCCAT-3’) primers (Liu et al., 1999), 12 μL of GoTaq Green Master Mix 2x (Promega, WI, USA), 4.5 μl of molecular water, and 40 ng of template DNA. The locus was amplified using a PCR cycle program of 94°C for 2 min, 40 cycles of 94°C for 40s, 58°C for 40s, and 72°C for 30s, and 72°C for 5 min. To visualize amplified PCR product using GelRed^®^ (Biotium), the products were electrophoresed on a 1.5% agarose gel and the amplified products sequenced in both directions using Sanger sequencing at Eurofins Genomics (https://eurofinsgenomics.com/en/home/). Base-score quality of the sequences were visually checked using Geneious Prime v. 2022.0.1 (https://www.geneious.com/) and identified to putative species through BLAST analysis in the National Center for Biotechnology Information (NCBI) database (https://www.ncbi.nlm.nih.gov/) and Fusarium-ID.v.3.0 (http://isolate.fusariumdb.org/blast.php) (O’Donnell et al., 2021).

A Bayesian inference phylogeny was constructed on the *rpb2* sequences with *Fusarium* reference strains to validate identity of *Fusarium* species. Details of the GenBank numbers of the reference strains used in the phylogeny are included in the **Supplementary Material** (**Supplementary Table 1**). The phylogeny indicated three potential *F. oxysporum* clades and one *F. solani* clade, but the phylogeny was constructed using a single locus and did not have enough signal to separate out the *F. oxysporum formae speciales* (**Supplementary Figure 2**). This warranted pathogenicity assays to be conducted to confirm whether the *Fusarium* isolates were able to cause disease on a susceptible common bean variety GLP2.

### 2.7 *Fusarium* pathogenicity test

**Figure 2:**
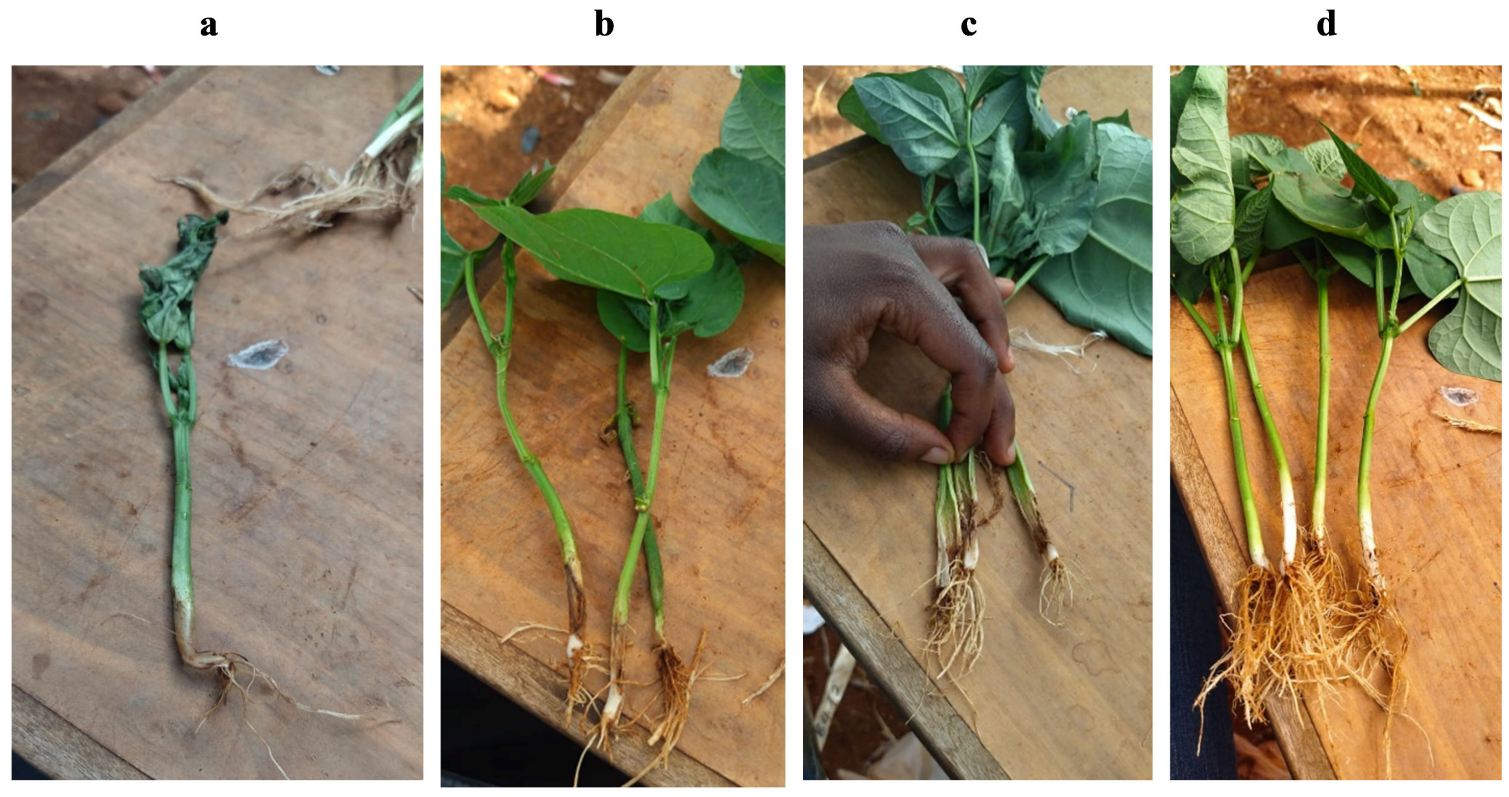
Infection of bean seedlings with *Fusarium* (the isolates from bean stems buried in soils from smallholder farms of western Kenya). Figure panels show the following: **(A)** wilted bean seedling grown in sterilized soil 5 days after stem inoculation with a conidial suspension of *F. oxysporum* isolated from the bean stem assay; **(B)** necrotic lesions on the lower stems of bean seedlings 10 days after inoculation with *F. solani*; **(C)** leaf wilting and browning of the xylem tissue (vascular discoloration) 10 days after inoculation with *F. oxysporum*; and **(D)** clean fibrous roots in the control treatment 20 days after germination.

To further validate interpretation of the visual *Fusarium* assay adapted from Furuya et al. (1999) and demonstrate the pathogenicity of the isolated strains, a common bean variety known to be susceptible to *Fusarium* a common bean variety (GLP2) was used to conduct pathogenicity assays. These assays were conducted on healthy bean seedlings 14 days after sowing in plastic pots (four plants per pot) containing ca. 500 g sterilized soil. *Fusarium* isolates grown on ½ PDA were used to make a conidial suspension that was then used to inoculate the healthy plants. To harvest the conidia, 1–5 ml of sterilized distilled water was placed onto the pure culture, which was then gently swirled and scraped. The conidial suspension was then filtered through two layers of muslin to remove mycelium. The suspension of 1 × 10^6^ cfu g^−1^ (colony forming unit/g) was then used as inoculum by pouring on a bruised lower stem of a healthy bean (10 days after germination), while sterile distilled water was used as an uninoculated control. Each isolate was inoculated on four replicates (pots) and these were randomly distributed on a table in a greenhouse. The pathogenicity tests were conducted twice under controlled conditions in a greenhouse, maintained at 25°C during the day and 19°C at night, at a relative humidity of ~80%. Pots were observed for 28 days after inoculation. Data was collected 5, 10, 15, 20, and 28 days post inoculation by randomly uprooting one plant per pot at each time point and collecting the following information: plant height, wilting (**Figures 2A, C**), vascular browning index (VBI) (**Figure 2B**), and dissection of the stem to check the length of browning along the inner stem. The rating scale for the VBI was as follows: 0 = no vascular discoloration; 1 = discoloration restricted to base of stem only; 2 = discoloration of the ‘internode 0’ (hypocotyl) region of the stem below the cotyledons; 3 = discoloration of stem above the cotyledons; 4 = complete vascular discoloration of stem; and 5 = plant death (Becerra Lopez-Lavalle et al., 2012).

To complete the confirmation that symptoms of infections were caused by the inoculated isolates, Koch’s postulates were conducted. The diseased bean plants were sampled, ensuring that samples were taken from the leading edge of the diseased area to avoid isolating secondary opportunistic invaders. The diseased samples yielded a range of fungi that morphologically were either *F. oxysporum*, *F*. *solani*, and *Rhizoctonia*. White bean stems, from beans grown in full darkness for 2 weeks and cut into 6 cm segments (as described in the *Fusarium* bioassay above) were employed to complete the Koch’s postulate analysis. Conidia from the isolated cultures were harvested by placing 1-5ml of distilled water onto the cultures and gently swirling and scraping them. The suspension of ca. 1 × 10^6^ cfu g^−1^ was used to inoculate sterilized soil (from less disturbed area at Kenya Agricultural and Livestock Research Organization, Kisumu station) and water holding capacity adjusted to 50%. Clean bean stems were then buried in the soils and covered in room temperature for 4 days. As described above, the number of reddish-brown lesions longer than 1 mm on the stems in each container and consistent with *Fusarium* damage were then counted.

### 2.8 Statistical analyses

To understand the potential of bioassays to provide a relative approximation of plant parasitic nematode populations compared to standard lab procedures, we used simple linear regression to assess correlations between bioassay-assessed nematode pressure and nematode communities assessed using lab extractions from roots (for *Meloidogyne*), and soil and roots (for *Pratylenchus*). We also used simple linear regression to explore relationships between the *Fusarium* bioassay and pathogenicity tests. Pairwise comparison of *Fusarium* isolate’s vascular browning index means was performed using the Tukey–HSD method, and statistical significance of differences between means was determined at *P* < 0.05. Impacts of the different management treatments across the eleven fields on pathogen pressure and soil physico-chemical variables were evaluated using ANOVA where a p-value ≤ 0.05 was considered significant. All comparisons considered treatment as a fixed variable and block as a random variable. Square root-transformations were used as needed to satisfy the ANOVA assumptions of homogeneity of variance and normality of residuals. All regression analyses and visualizations were conducted using R (version 4.0.4; and “ggplot2”, “dplyr”, and “ggpubr” packages), while ANOVA comparisons were done using JMP (version 15.0.0).

## 3 Results

### 3.1 Comparison of bioassay vs. lab assessment of nematodes

**Figure 3:**
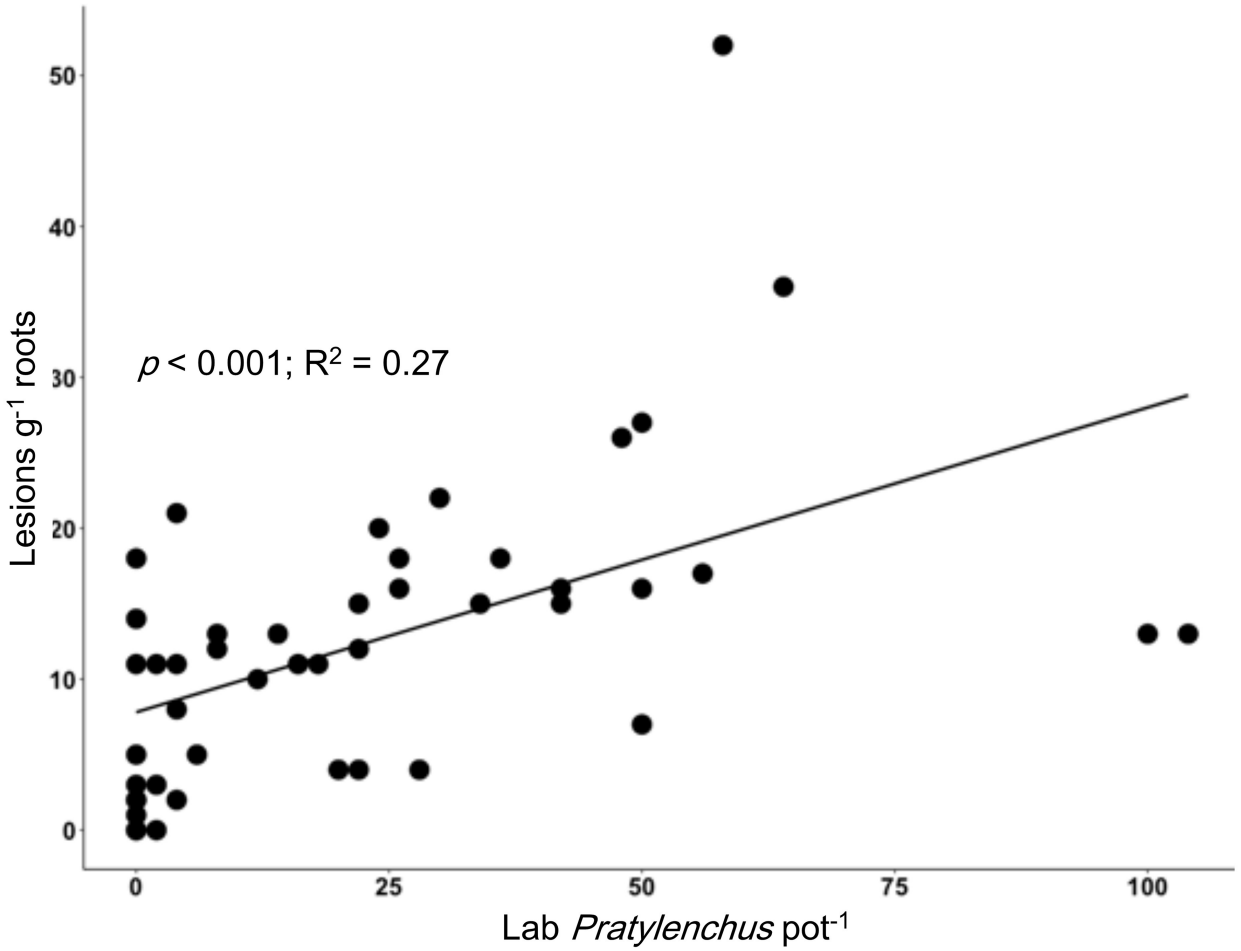
Relationship between root lesion density in the nematode bioassay using soybean as a bait plant (y-axis, lesions g^-1^ root) and a standard laboratory assessment of *Pratylenchus* per pot (x-axis, individuals per pot in roots and soil).

We identified various nematode taxa from the soils and soybean roots analyzed in the lab, including multiple PPN taxa. From soybean roots, we extracted *Pratylenchus, Tylenchorynchus, Helicotylenchus, Scutellonema*, and *Meloidogyne*, while in soils (of the same pots) we observed *Meloidogyne, Trichodorus, Aphelenchoides, Dorylaimodes, Pratylenchus, Helicotylenchus, Scutellonema*, and X*iphinema*. The number of lesions found on soybean roots was positively correlated with the *Pratylenchus* extracted from the roots (*p* < 0.001; R^2^ = 0.27; **Figure 3**). Significant relationships were also observed for the number of lesions on growing soybean roots and *Pratylenchus* abundance in soil alone (*p* = 0.049; R^2^ = 0.09) as well as in soil and roots combined (*p* = 0.026; R^2^ = 0.12). We note that there were a number of cases where lesions were observed on soybean roots, but no *Pratylenchus* spp. were recovered in the sample. The fact that the soil used hosted other PPN (*Trichodorus, Aphelenchoides, Dorylaimodes, Helicotylenchus, Scutellonema*, and *Xiphinema*) and soil-borne pathogens such as *Fusarium* and *Pythium*, could explain the high variation as these pathogens can work as a complex.

**Figure 4:**
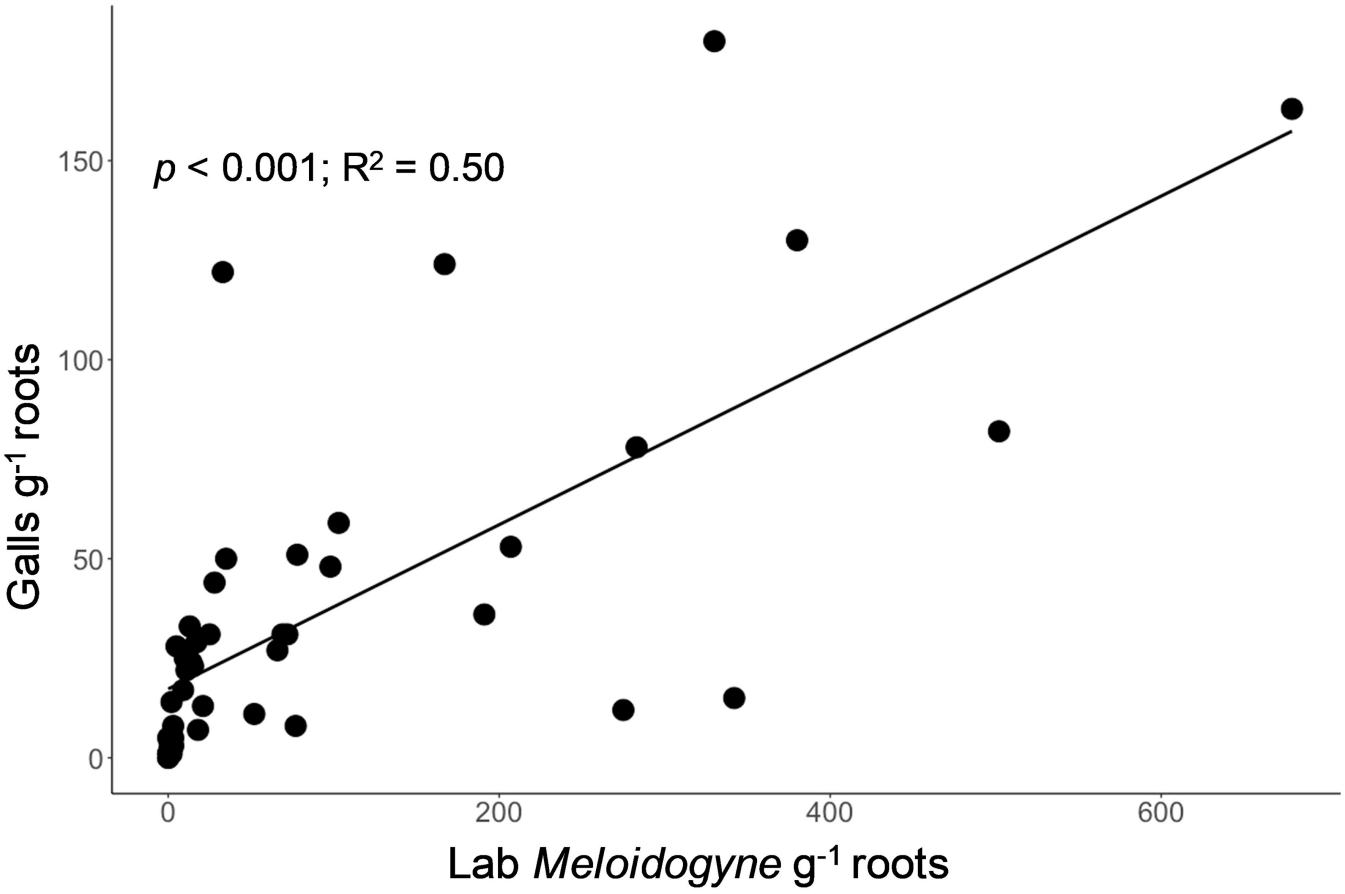
Relationship between the visual assessment of the number of galls on lettuce root (y-axis, galls g^-1^ root) and a standard laboratory assessment of *Meloidogyne* nematodes extracted from lettuce root (x-axis, individuals g^-1^ root) for assessment of root-knot nematode bioassay.

Galls assessed on lettuce roots were strongly related to the number of *Meloidogyne* nematodes (second-stage juveniles (j2s) and adult) extracted from lettuce roots in the lab (p < 0.001; R^2^ = 0.50, **Figure 4**). In addition to *Meloidogyne*, *Trichodorus, Pratylenchus, Helicotylenchus, Scutellonema, Xiphinema, Hoplolaimus*, and *Hemicyclophora* were found extracts from lettuce roots in the lab, indicating a wide variety of nematode species are likely causing damage on smallholder farms in our study region. In a few cases, lettuce roots were rotten or beginning to rot by the time of visual root assessment, which made it difficult to check for root galls (hence the galls were not counted).

The total number of plant parasitic nematode taxa extracted from all our samples were 5,576 *Scutellonema*, 3,384 *Meloidogyne*, 1,228 *Helicotylenchus*, 1,035 *Dorylaimodes*, 810 *Xiphinema*, 572 *Pratylenchus*, 455 *Aphelenchoides*, 151 *Tylenchorynchus*, 87 *Trichodorus*, 20 *Hoplolaimus*, and 10 *Hemicyclophora*.

### 3.2 *Fusarium* isolates and pathogenicity

**Figure 5:**
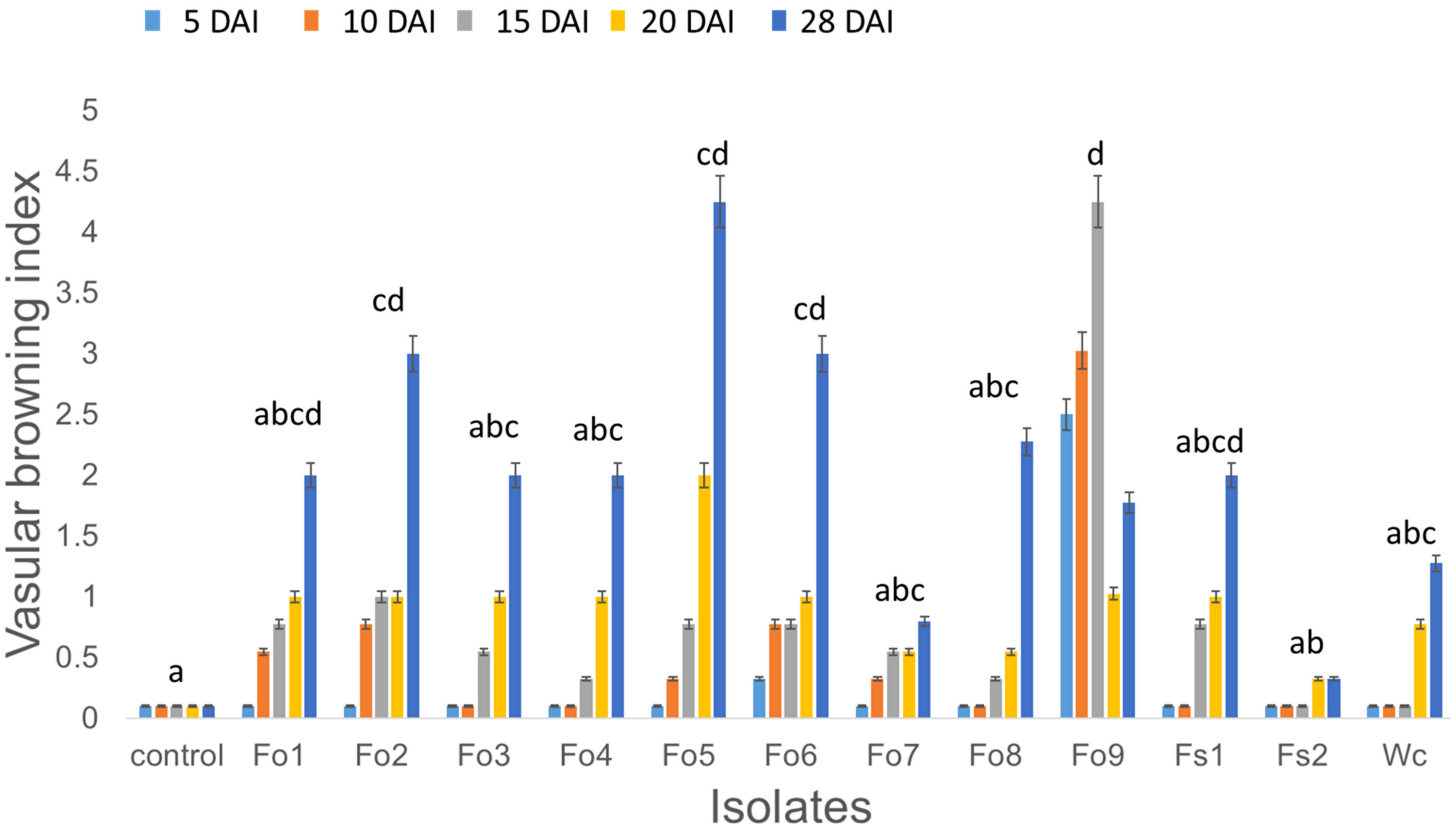
The effect of *Fusarium* inoculation on average vascular browning index of healthy bean seedlings at: 5, 10, 15, 20, and 28 days after inoculation (DAI). Measurements were taken every 5 days after beginning inoculation by randomly sampling a bean plant from each pot inoculated with individual isolates: *Fusarium oxysporum* type 1 (Fo1) to type 9 (Fo9), *Fusarium solani* 1 (Fs1) and type 2 (Fs2), and *Waitea circinata* (Wc). The isolates were identified to species level by molecular analysis (**Supplementary Table 1**; **Supplementary Figure 2**). Letters represent pairwise comparison of average vascular browning index across the different isolates, and bars with the same letter are not significantly different at *p* < 0.05.

Based on both morphological and molecular methods, nine *F. oxysporum*, two *F. solani*, and one *Waitea cincinata* isolates were identified (**Supplementary Table 1**; **Supplementary Figure 2**). The pathogenicity test indicated that all nine isolates had the potential to cause wilting, vascular browning, rotting, stunting, and/or root necrosis, relative to controls inoculated with distilled sterile water (**Figure 2**). These pathogens appeared to infect root vascular tissues, leading to browning and rotting of roots, which affected general plant health. The virulence levels of these pathogens also appeared to vary (**Figure 5**), with *F*. *oxysporum* (Fo5 (*p* = 0.012) and Fo9 (*p* < 0.001) having the highest browning index and *F. solani* (Fs2) having the least, compared to control. Despite the few isolates used in pathogenicity test, lesions assessed on bean stems four days after burying them in soil were slightly correlated with select isolate’s ability to cause browning in healthy bean inner stem (**Supplementary Figure 1**).

### 3.3 Management impacts on soil health parameters

The three soil treatments tested (i.e., manure, synthetic fertilizer, control) did not demonstrate significant effects on the measured soil health parameters (e.g., POXC, aggregate stability pH; **Table 1**). However, *Pythium*, *Fusarium*, and root-knot nematodes differed significantly among the soil amendment treatments. *Pythium* pressure was less prevalent (35% less; *p* = 0.02) in plots receiving FYM compared to the no-input control, while *Fusarium* lesions were 23% higher (*p* = 0.01) in the FYM treatment, compared to no-input control (**Table 1**). At the same time, root galls associated with *Meloidogyne* were three times more abundant in the plots receiving synthetic fertilizer (DAP) compared to control (p < 0.001), which had the fewest number of galls. No significant differences were observed for Pratylenchus between treatments. While there were no significant differences in assessed soil health properties, it is important to note that various soil properties (e.g., POM, aggregate stability, and pH) were, on average, higher in FYM compared to DAP and the control (**Table 1**).

**Table 1:** Soil health parameters and soil-borne pathogens tested across three treatments [control, di-ammonium phosphate (DAP), and farmyard manure (FYM)] applied for two long-rainy seasons in eleven farms across three locations in western Kenya.

	Control	DAP	FYM	P values
**P_avail_ (mg P kg^-1^)**	4.59 (0.73)	5.55 (1.04)	4.09 (0.56)	0.41
MWD (µm)^*^	757.6 (38.0)	776.50 (26.5)	810.50 (40.6)	0.550
**POXC (mg C kg^-1^)**	469.8 (53.7)	516.32 (46.6)	511.23 (56.5)	0.780
**pH**	5.70 (0.11)	5.73 (0.09)	5.80 (0.10)	0.740
**POM (mg kg^−1^)**	130 (30)	120 (30)	140 (20)	0.780
**Org C (g kg^−1^)**	1.98 (0.15)	2.01 (0.15)	1.97 (0.15)	0.980
***Pythium* (colonies plate^-1^)**	5.41 (0.53) a	5.18 (0.51) a	3.50 (0.49) b	**0.020**
***Fusarium* (lesions stem^-1^)**	17.32 (1.80) ab	13.14 (1.65) b	21.23 (1.72) a	**0.010**
***Meloidogyne* (knots g^-1^ root)**	27.00 (6.11) b	68.02 (13.3) a	22.21 (10.3) b	**< 0.001**
***Pratylenchus* (lesions g^-1^ root)**	30.80 (10.29)	9.35 (1.97)	11.93 (4.77)	0.150

*P_Avail_, available P; MWD, mean weight diameter; POXC: Permanganate oxidizable carbon; POM, Particulate organic matter; Orc C, organic carbon. Standard errors are presented to the right of each mean in parentheses. Means followed by different letters indicate significant differences between treatments (ANOVA, p < 0.05), with significant p-values denoted in bold.

### 3.4 Nematode communities in relation to nutrient input treatments

Assessment of soil nematode communities indicated large differences in the number of free-living nematodes across the three nutrient input treatments. We note that three genera of predators (*Labronema, Mononchus, and Discolaimodes)*, five bacterivores (P*rismatolaimus, Acrobeles, Cephalobus, Eucephalobus, and Rhabditis)*, and two fungivores (*Filenchus and Aphelenchus*) were identified from soybean bioassay soil samples used to assess lesion nematodes. Bacterivores and fungivores were 200% and 75% more abundant in FYM soils, respectively, compared to the no-input controls (**Table 2**). Notably, the lowest number of bacterivores and fungivores were recorded in DAP amended soils. The proportion of PPN in the whole soil nematode community (and indicator of PPN pressure) was lowest in the FYM treatment, and highest in the DAP treatment (*p* = 0.003).

**Table 2:** Average nematode abundances extracted from incubated soils across three treatments [control, di-ammonium phosphate (DAP), and farmyard manure (FYM)] applied for two long-rains seasons in 11 farms in three locations in western Kenya.

	Average			Treatments
	Control	DAP	FYM	P-values
**Bacterivores**	1554 (188.7) b	123 (144.7) b	3580 (484.9) a	**< 0.001**
**Fungivores**	109 (18.7) b	84 (15.6) b	186 (22.8) a	**0.021**
**Predators**	35 (6.2)	47 (7.0)	31 (4.3)	0.451
**Plant parasitic nematodes (PPN)**	110 (11.4) b	246 (21.6) a	85 (9.1) b	**< 0.001**
PPN as proportion of total^*^	0.12 (0.0) b	0.19 (0.04) a	0.07 (0.0) b	**0.003**

* the proportion of plant parasitic nematodes relative to all nematodes recovered. Standard errors are presented to the right of each mean in parentheses. Means followed by different letters indicate significant differences between treatments (ANOVA, p = 0.05), with significant p-values denoted in bold.

## 4 Discussion

This study sought to evaluate the performance of simplified tests for soil-borne diseases and nematodes and the ability of these tests to assess the impact of common nutrient inputs on soil root pathogens and relationships with key soil health parameters. Our results suggest that the bioassays examined here offer considerable promise to provide farmers and local technicians with more accessible techniques to evaluate levels of *Fusarium, Pythium, Meloidogyne*, and *Pratylenchus* in soil. Specifically, the bioassay indicators for PPN (i.e., lesions, galls) were found to correlate moderately well with the lab-assessed nematode abundances (**Figures 3**, **5**), while the recovered *Fusarium* in the plant stem assay caused disease, highlighting the success of the *Fusarium* bioassay (**Figure 5**). Bioassays using susceptible crops have historically been used to assess levels of various soil pathogens. These include the use of potato to quantify *Pythium aphanidermatum* in soil (Stanghellini and Kronland, 1985), eggplant to assess *Verticillium dahliae* (Nagtzaam et al., 1997), cotton to assess *Fusarium* wilt disease (Becerra Lopez-Lavalle et al., 2012), and spinach to predict risks of *Fusarium* wilt (Gatch and du Toit, 2015). Bioassays are useful quantitative tools that can be used to evaluate changes in a system and harmful effects of management on different factors (Terekhova, 2011). They are also valuable in that they integrate biological processes over time and provide a measure that is directly relevant to plant growth. We note that the bioassays assessed in this study are easy to follow, cost-effective, rapid, and visual, making them potentially valuable tools for smallholder systems where soil-borne pathogen pressure is high and formal laboratories are inaccessible. In addition, the assays can provide research organizations working with smallholder farmers with essential tools to train the farmers who might not have skills to identify soilborne pathogen symptoms.

### 4.1 Comparison of bioassay vs. lab assessment of nematodes

*Meloidogyne* and *Pratylenchus* were found in all the soils from the eleven farms, across three different locations in Nandi County. Laboratory analyses also revealed the presence of other PPN taxa (*Scutellonema, Helicotylenchus, Dorylaimodes, Xiphinema, Aphelenchoides, Tylenchorynchus, Trichodorus, Hoplolaimus*, and *Hemicyclophora*) in all farms. This is in agreement with other reports that, although *Meloidogyne* and *Pratylenchus* species are likely the most important nematode pests in SSA, the typical crops and local conditions are associated with many PPN, which are part of a larger, complex nematode community (Coyne et al., 2018; Sikora et al., 2018). Understanding the relative importance of nematode species existing in complex communities is difficult, especially since they have varying generation periods and may thrive under different environmental conditions that vary seasonally (Luc et al., 2005).

*Pratylenchus* spp. are categorized as migratory endoparasites, mostly feeding and reproducing within the root system but sometimes feeding on the root surface without entering the root tissue and can also be found in soils surrounding roots. *Pratylenchus* enter and feed on plant tissue, secreting cell-wall degrading enzymes and leaving brown elongated lesions that eventually become necrotic areas (Gugino et al., 2006). While these signs can be visually observed, absolute association of brown lesions with *Pratylenchus* only, especially in soils infested with other PPNs can be challenging. We note that the bioassay used here lasted for 3 weeks and other PPN signs may not have fully developed. Coyne et al. (2018) suggests that crops grown in SSA are associated with remarkably complex nematodes communities, and it may be difficult to evaluate the relative pathogenicity of individual species. Therefore, the high variance observed (R^2 =^ 0.27) could be associated with the presence of other soil-borne pathogens that cause necrotic lesions on soybean roots, working as a complex. However, despite the variability observed here the relationship had high statistical significance (*p* < 0.001; **Figure 5**) showing that counting lesions in the bioassay can reasonably assess the pressure of *Pratylenchus*. In previous research, methods used in this study were tested with commercial vegetable growers in New York, and results demonstrated that the number of lesions developed on the soybean roots corresponds to the relative lesion nematode infestation level in the soil (Gugino et al., 2009).

Many zero counts for *Meloidogyne* abundance in the lab assessments contributed significant variability and complicated the ability of our bioassay to predict *Meloidogyne* abundance. In some cases, using the bioassay, many galls were identified, but the lab extraction found zero root-knot nematodes in the J2s growth stage. This could be explained by the fact that the adult sedentary stage of *Meloidogyne* females involve feeding, swelling into pear-shape, and producing egg masses (500 to 1000; surrounded by a gelatinous matrix) on the root surface (Tapia-Vázquez et al., 2022; Vilela et al., 2023), in which these swellings could be easily counted as root galls. If root extraction is done before the eggs hatch to j2s, lab analyses would register zero *Meloidogyne*. We suspect that this relative “over counting” of reproducing females as “root knots” may be capturing future generations of root-knot nematodes, since these females will likely hatch hundreds of infective J2s, potentially making the bioassay relatively powerful as a method to assess crop risk from *Meloidogyne*. However, further research is needed to confirm this idea and provide a clearer interpretation for the gall counts.

### 4.2 Management impact on nematode communities

There were higher number of fungivores and bacterivores in the FYM plots suggesting that a greater level of labile carbon inputs in manure-treated plots may play a role in developing more balanced nematode communities in this treatment. Farmyard manure is known to improve physical and chemical properties of soil (Riegel and Noe, 2000; Liu et al., 2021; Karuri, 2022) making them more conducive for a diverse array of soil microbes, including bacteria and fungi. By enhancing bacterial and fungal decomposers, this supports greater populations of bacterial and fungal feeding nematodes (Rosskopf et al., 2020). Given the lack of differences in predatory nematodes between treatments, it appears that the high number of bacterivores and fungivores, and low number of PPN in manure-amended soils could be as result of parasitism of PPN by soil microbes. Animal manure is known to support proliferation of antagonistic fungi and bacteria, which can then reduce PPN (Bailey and Lazarovits, 2003; Oka, 2010). Although the impact that organic amendments have on soil nematodes and microbial communities can be quite complex, possible mechanisms for the suppression of nematodes include release of compounds such as ammonia and fatty acids that can be nematicidal, enrichment of antagonistic organisms, change in soil physiology, or plant tolerance and resistance improvement (Oka, 2010). However, contrasting effects of manure on PPN have been reported, suggesting the need for further assessment of manure effectiveness to assist in managing PPN across variable farm field conditions in SSA (Karuri, 2022). While the recent establishment of the trials did not permit detection of significant impacts on soil health parameters (e.g., pH, POM, and MWD) in the FYM treatment, compared to DAP and control, we suspect that organic matter amendments are quite important for soil health and that the changes may be more pronounced if organic amendments are used for longer periods. This idea is supported by results from Ayuke et al. (2011) who evaluated long-term impacts of amending soil with organic inputs vs. synthetic fertilizers on soil chemistry and biology in central Kenya and found that FYM significantly improved C and N pools, as well as biological activity and diversity (i.e., macrofauna abundance).

### 4.3 Management impact on *Pythium* and *Fusarium*

The significantly lower abundance of *Pythium* under the FYM-amended soil is likely related to the high inputs of organic matter in the FYM treatment that likely lower pathogenic *Pythium* development by promoting competition and antagonism by other organisms (Le et al., 2014). Several modes of action are involved in the activity of manure; these include the release of allelochemicals generated by consequent microbial decomposition (Bünemann et al., 2006) and increase in soil organic matter that improve water retention, create microhabitats less conducive for soil pathogens or possibly introducing biocontrol capabilities such as enhancement of organisms that are antagonists, competitors, or parasitic against soil-borne organisms (Rosskopf et al., 2020).

In contrast to *Pythium*, *Fusarium* appeared to be significantly higher in plots amended with FYM. While this may seem unusual, the broad review and discussion by Alabouvette et al. (2009) indicates that the diverse species of *Fusarium* are complex, and with FYM application the diversity of *Fusarium* spp. might have been greater. Moreover, all *Fusarium* strains feed on available carbon, which was more readily available in the FYM treatment. Importantly, Alabouvette et al. (2009) discuss that both pathogenic and non-pathogenic *Fusarium* strains have the ability to colonize the root surface and to penetrate the root, in a similar manner. However, antagonism through either competition for infection sites between pathogenic and nonpathogenic strains or parasitism, are common and the main mechanisms of biocontrol by the non-pathogenic strains. In our study, the many lesions that developed on bean stems were observed in soils amended with manure, suggesting a high level of *Fusarium* in those soils. This suggests that the bioassay used in our study provides an indication of possible level of infection from pathogenic *Fusarium*, but that the actual impact on plant growth is mediated by soil health and soil fertility status on both pathogens and plant growth. So, incorporation of organic inputs such as FYM will enhance a number of soil properties (e.g., soil pH, water holding capacity, aeration, carbon and nutrient availability), creating a more conducive environment for soil-borne pathogens, including *Fusarium*, to survive. At the same time, these conditions often allow plants to grow more vigorously and potentially defend themselves better against pathogens. The opposite would be the case in soil with poor soil health and fertility, which will stress the crops (nutrients, drainage and compaction effects e.g.) and limit root growth (Medvecky and Ketterings, 2009), increasing crop vulnerability to pathogenic *Fusarium*. We suggest the need for additional research that examines *Fusarium* presence links to pathogenicity across a range of soil health contexts.

### 4.4 Implications and potential applications

The adapted bioassays evaluated here offer great potential for smallholder farmers, with the help of extension and field officers, to efficiently assess and manage soil-borne pathogens. Soil analysis using these bioassays could be combined with other soil health tests prior to planting, to provide farmers with valuable insight on the potential disease pressure and possible management interventions to reduce pathogen severity. Additionally, farmers and local technicians could use soil bioassays to evaluate the effectiveness of alternative or novel management practices designed to reduce pathogen pressure. The visual nature of the bioassays considered here allow for participatory root assessment to be done collaboratively between farmers and extension officers, thus helping farmers to improve knowledge and awareness of soil-borne pathogens and facilitate the development of practices that lessen their impact. A better understanding of the problem would likely support the adoption of specific practices that show promise to lower soil-borne pathogen presence and associated threats. Despite their great promise, additional research is needed to better understand how the bioassays evaluated here relate to crop yields, and more specifically, to economic threshold levels for the management of *Fusarium*, *Pythium*, *Pratylenchus* and *Meloidogyne*. We suspect that in many cases a pathogen maybe present in a system, but that the cost of implementing alternative management practices or other pest control measures, likely exceeds the potential cost of doing nothing; alternatively, high existing levels soil fertility and soil health generally may moderate impacts of disease on yield as discussed above.

Beyond demonstrating the potential of bioassays to inform about pest and disease issues in smallholder farming systems, our research offers some important insight about management strategies to lessen the impact of soil-borne pathogens. Specifically, our findings suggest that farmers experiencing disease symptoms linked to *Pythium* and PPN (e.g., seeds and seedling rots, stem collapse, damping-off, yellowing patches, root knots, or necrotic lesions) would benefit from more frequent application of FYM. Other strategies might include growing less susceptible crops, and diversifying cropping systems through intercropping, more complex rotations, or agroforestry, all practices known to enhance general soil health status and reduce crop damage (Peiris et al., 2020; Shen et al., 2023; Yu et al., 2023). Given the heterogeneous nature of smallholder farming environments and management (e.g., Nyamasoka-Magonziwa et al., 2021), we suspect that the most effective strategies likely vary with context. As such, we suggest that the bioassays evaluated here can help researchers, field officers, and farmers to better map and understand the factors that drive soil-borne pathogen pressure and impacts on crops, and thus help guide future research to address challenges in smallholder farming systems.

## 5 Conclusions

Smallholder farming systems of SSA are often characterized by continuous cultivation and limited crop diversity, thus contributing to favorable conditions for survival and multiple generations of soil-borne pathogens. Our findings confirm the existence of major soil-borne pathogens across the entire study area and suggest that simplified soil bioassays offer a valuable tool for smallholder farmers, in collaboration with local extension agents, to better evaluate and understand pathogen inoculum pressure on-farm. Furthermore, our results show that organic amendments such as FYM promote more robust soil communities and potentially suppression of PPN (as indicated by the community composition of free-living nematodes), likely as a result of improvements to C availability and overall soil health.

## Data Availability

The datasets presented in this study can be found in online repositories. The names of the repository/repositories and accession number(s) can be found in the article/**Supplementary material**.
