# Supplementary figures and images for: Assessing performance of simplified bioassays for soil-borne pathogens in smallholder systems of western Kenya
Source: Front Plant Sci. 2024 Aug 15;15:1389285. doi: 10.3389/fpls.2024.1389285 (PMC11360875; doi:10.3389/fpls.2024.1389285)

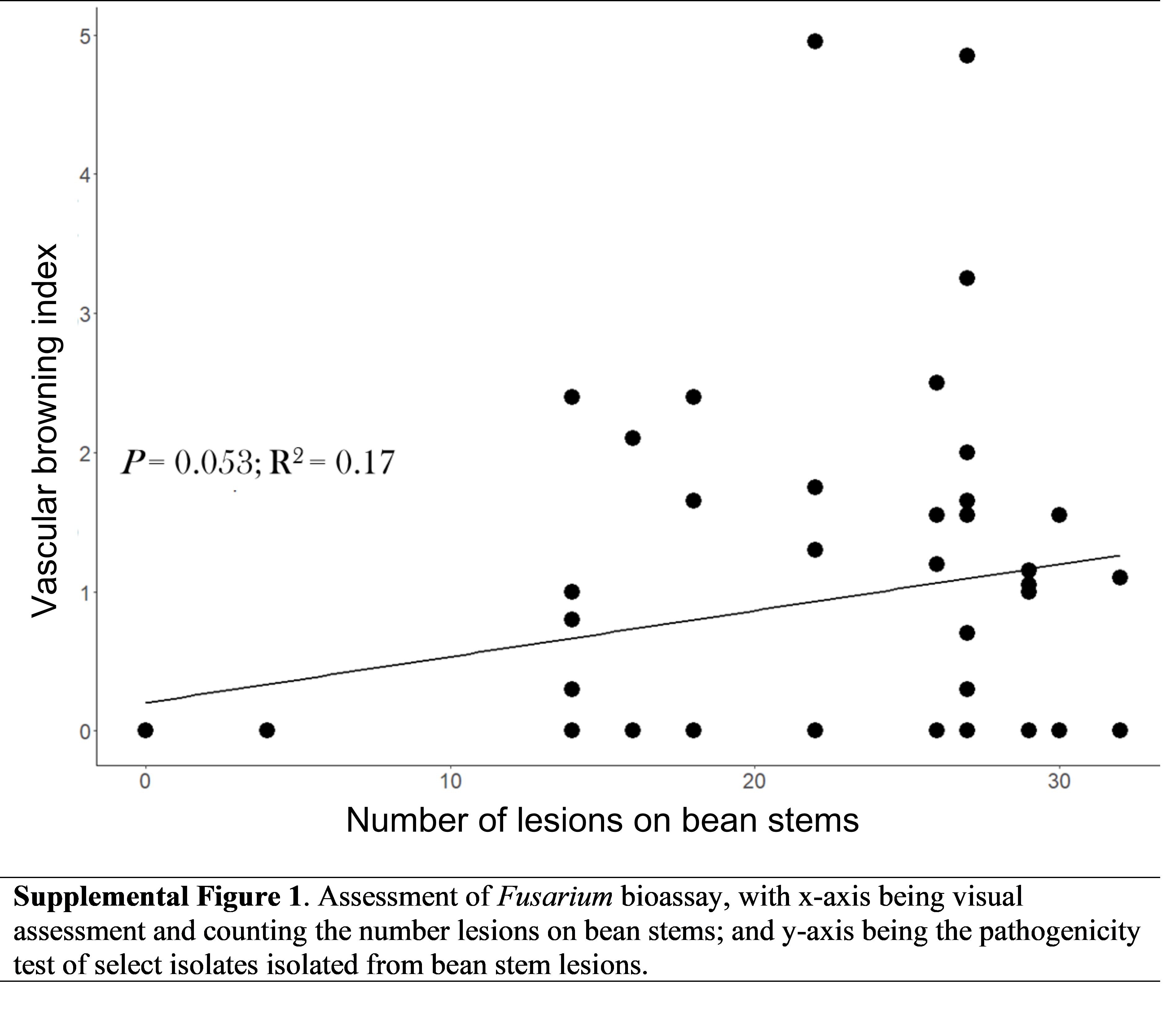

Supplement: Supplementary file 1 [file Image1.jpg]

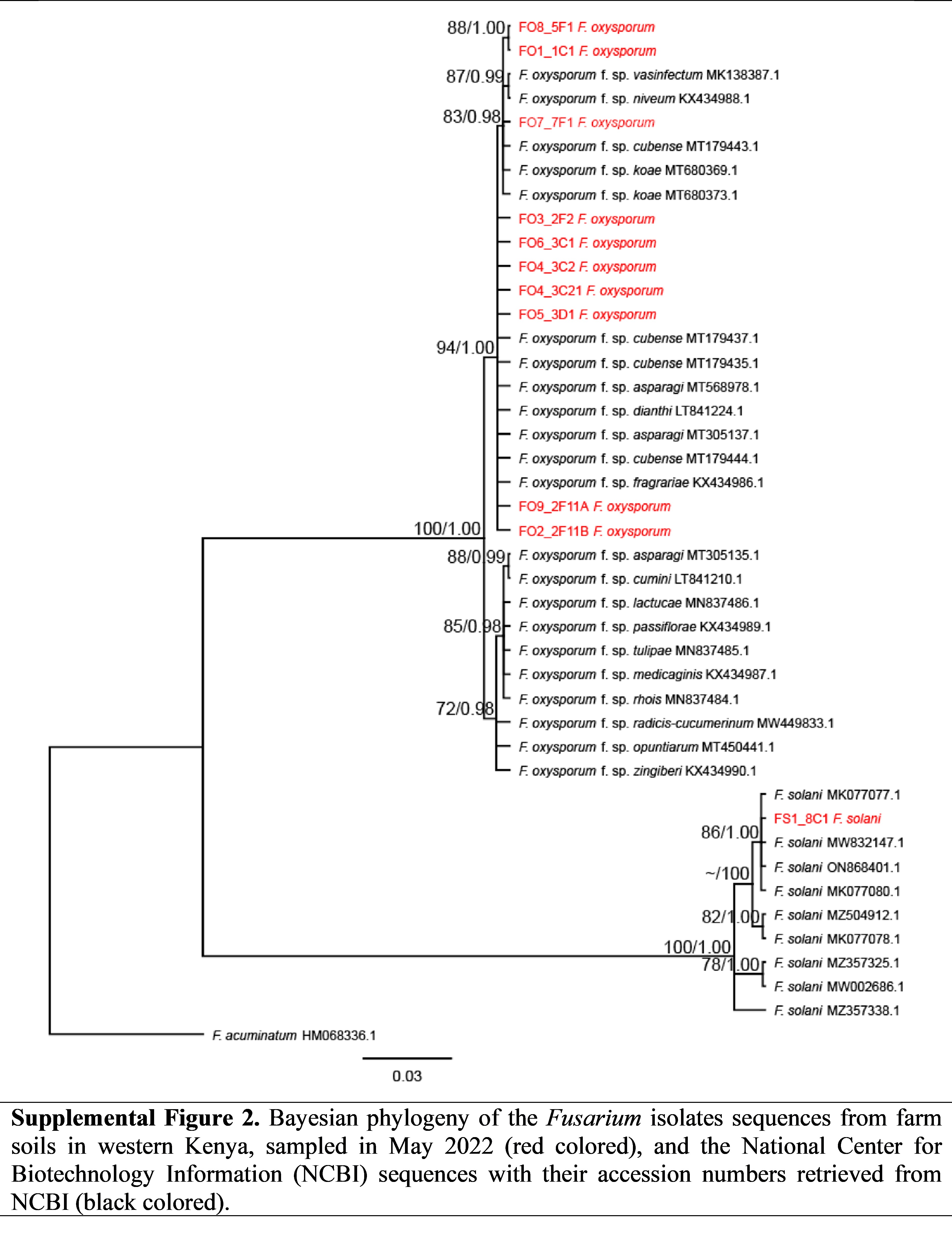

Supplement: Supplementary file 2 [file Image2.jpg]
