# Supplementary material for: Assessing performance of simplified bioassays for soil-borne pathogens in smallholder systems of western Kenya
Source: Front Plant Sci. 2024 Aug 15;15:1389285. doi: 10.3389/fpls.2024.1389285 (PMC11360875; doi:10.3389/fpls.2024.1389285)
Supplement: Supplementary file 3 [file Table1.pdf]

Supplemental Table 1. Accession numbers for *Fusarium* and *Waitea* isolates from this study and reference strains from National Center for Biotechnology Information (NCBI) used in phylogenic analysis.

Isolate_ID	Organism	*Forma specialis*	GenBank accession number	Isolate_Origin
FO7_7F1	*Fusarium oxysporum*		OR355479	This study
FO1_1C1	*Fusarium oxysporum*		OR355470	This study
FO3_2F2	*Fusarium oxysporum*		OR355471	This study
FO8_5F1	*Fusarium oxysporum*		OR355478	This study
FO2_2F11B	*Fusarium oxysporum*		OR355473	This study
FO4_3C2	*Fusarium oxysporum*		OR355475	This study
FO5_3D1	*Fusarium oxysporum*		OR355477	This study
FO4_3C21	*Fusarium oxysporum*		OR355476	This study
FO6_3C1	*Fusarium oxysporum*		OR355474	This study
FO9_2F11A	*Fusarium oxysporum*		OR355472	This study
FS1_8C1	*Fusarium solani*		OR355480	This study
Wc_4F2A	*Waitea circinata*		OR355481	This study
Wc_4F2B	*Waitea circinata*		OR355482	This study
NRRL 54218	*Fusarium acuminatum*		HM068336.1	Reference
BRIP39299	*Fusarium oxysporum*	*zingiberi*	KX434990.1	Reference
DB18AGO01	*Fusarium oxysporum*	*opuntiarum*	MT450441.1	Reference
BRIP28044	*Fusarium oxysporum*	*passiflorae*	KX434989.1	Reference
BRIP5189	*Fusarium oxysporum*	*medicaginis*	KX434987.1	Reference
PD01504750896	*Fusarium oxysporum*	*lactucae*	MN837486.1	Reference
CBS 220.49	*Fusarium oxysporum*	*rhois*	MN837484.1	Reference
CBS 242.59	*Fusarium oxysporum*	*tulipae*	MN837485.1	Reference
F11	*Fusarium oxysporum*	*cumini*	LT841210.1	Reference
GR_FOA168	*Fusarium oxysporum*	*asparagi*	MT305135.1	Reference
FRCKSU17	*Fusarium oxysporum*	*radicis-cucmerinum*	MW449833.1	Reference
CAV3484	*Fusarium oxysporum*	*cubense*	MT179437.1	Reference
RBH3	*Fusarium oxysporum*	*vasinfectum*	MK138387.1	Reference
NTP-Dc36955	*Fusarium oxysporum*	*niveum*	KX434988.1	Reference
CAV3130	*Fusarium oxysporum*	*cubense*	MT179443.1	Reference
44	*Fusarium oxysporum*	*koae*	MT680373.1	Reference
166	*Fusarium oxysporum*	*koae*	MT680369.1	Reference
CAV3128	*Fusarium oxysporum*	*cubense*	MT179444.1	Reference
GR_FOA230	*Fusarium oxysporum*	*asparagi*	MT305137.1	Reference
	Fusarium oxysporum	*dianthi*	LT841224.1	Reference
BRIP53860	*Fusarium oxysporum*	*fragariae*	KX434986.1	Reference
CAV	*Fusarium oxysporum*	*cubense*	MT179435.1	Reference
MA_FOA24	*Fusarium oxysporum*	*asparagi*	MT568978.1	Reference
KARE233	*Fusarium solani*		MK077077.1	Reference
KARE221	*Fusarium solani*		MK077080.1	Reference
BMU 03270	*Fusarium solani*		MW832147.1	Reference
JZB3110248	*Fusarium solani*		ON868401.1	Reference
KARE398	*Fusarium solani*		MK077078.1	Reference

XG1 bai	*Fusarium solani*	MZ504912.1	Reference
NsPed1	*Fusarium solani*	MW002686.1	Reference
CHS8	*Fusarium solani*	MZ357325.1	Reference
TNM31R	*Fusarium solani*	MZ357338.1	Reference
